# Characteristics and factors associated with mortality in palliative patients visiting the Emergency Department of a large tertiary hospital in Thailand

**DOI:** 10.1186/s12904-022-01009-z

**Published:** 2022-06-27

**Authors:** Apichaya Monsomboon, Trisuchon Chongwatcharasatit, Pratamaporn Chanthong, Tipa Chakorn, Nattakarn Prapruetkit, Usapan Surabenjawong, Chok Limsuwat, Wansiri Chaisirin, Onlak Ruangsomboon

**Affiliations:** 1 Department of Emergency Medicine, Faculty of Medicine, Siriraj Hospital, Mahidol University, 2 Wanglang Road, Bangkoknoi, Bangkok, 10700 Thailand; 2 Siriraj Palliative Care Center, Mahidol University, 2 Wanglang Road, Bangkoknoi, Bangkok, 10700 Thailand

**Keywords:** Palliative care, Palliative patient, Advance care plan, Emergency department visit

## Abstract

**Background:**

The characteristics and outcomes of palliative patients who visited the Emergency Department (ED) in Thailand, a country in which no standard palliative care system existed, have not been comprehensively studied. We aimed to report the characteristics of ED palliative patients and investigate factors associated with mortality.

**Methods:**

A prospective observational study was conducted at Siriraj Hospital, Bangkok, Thailand, between March 2019 and February 2021 by means of interviewing palliative patients and/or their caregivers and medical record review. Palliative patients with either incurable cancer or other end-stage chronic diseases were included.

**Results:**

A total of 182 patients were enrolled. Their mean age was 73 years, 61.5% were female, and 53.8% had incurable cancer. Of these, 20.3% had previously visited the palliative clinic. Approximately 60% had advanced directives, 4.9% had a living will, and 27.5% had plans on their preferred place of death. The most common chief complaint was dyspnea (43.4%), and the main reason for ED visits was ‘cannot control symptoms’ (80%). At the ED, 17% of the patients had been seen by the palliative care team, and 23.1% died. Although 51% were admitted, 48.9% could not survive to discharge. Cancer, having received morphine, a palliative performance scale > 30, and ED palliative consultation were independently associated with hospital mortality.

**Conclusion:**

The recognition and utilization of palliative care were largely inadequate, especially for non-cancer patients. An improvement and promotion in the palliative care system from the ED through home care are mandatory to improve the quality of life of palliative patients.

**Supplementary Information:**

The online version contains supplementary material available at 10.1186/s12904-022-01009-z.

## Introduction

With our move toward an ageing society, more people have developed chronic and incurable diseases. Patients with these advanced illnesses usually receive supportive and symptomatic palliative treatment at home or other nursing residences. However, during the last period of their lives, they generally visit the Emergency Department (ED) more frequently [1–3]. Some of these visits are avoidable by integrating appropriate and high-quality palliative treatment into the standard of care, while many others are not. Previous studies have reported variable rates of unavoidable ED visits ranging from 45 to 95% with various presenting symptoms, such as pain, respiratory and gastrointestinal problems, fatigue, and altered mental status [2, 4–8]. Although unavoidable visits may occur due to unprecedented symptoms and conditions for which patients truly require urgent care, most are still a result of suboptimal palliative treatment. Advance care planning is often initiated too late in the disease trajectory. Also, patients, families, and healthcare providers are often not educated well enough and are thus not timely prepared for expected future problems. Consequently, these patients have to visit and be admitted to the ED and the hospital despite their wishes [9, 10]. These visits are an indicator of poor end-of-life care and ineffective palliative care systems, and can also result in caregiver burden and ED crowding [7, 11, 12]. Many studies have also emphasized the significant amount of ED resources utilized by the palliative population [4, 6, 8, 13]. Moreover, some patients could not survive to discharge and had to spend their true end-of-life in the hospital against their wishes. According to previous studies, patients particularly at higher risk of in-hospital mortality were those presenting with dyspnea, altered mental status and weakness/fatigue, and patients with gastrointestinal tract cancer, hypoalbuminemia, and poor performance status [14, 15].

In Thailand, the National Health Act of Thailand launched in 2007 has highlighted the right of the people to make a living will to refuse health services that are provided merely to prolong their terminal stage of life [16]. After this act was published, the Ministry of Public Health has emphasized policies relating to palliative care starting from the primary care system to the hospitals nationwide. However, there are still no governmental hospices or standard systematic long-term care services for palliative patients up until now. With such a distinctive scenario, the characteristics of palliative patients visiting the ED in Thailand may differ significantly from other settings with more standardized practices and systems. Also, understanding the causes and consequences of these visits, as well as factors associated with poor outcomes, may facilitate future development of appropriate and effective palliative care services. Therefore, this study was conducted to investigate the characteristics of palliative patients who visited the ED and the causes of their visits. We also aimed to analyze factors associated with mortality in the ED and at hospital discharge in these patients.

## Methods

### Study design and setting

This prospective observational study was conducted between March 2019 and February 2021 at the ED of Siriraj Hospital, the largest tertiary university hospital in Bangkok, Thailand, with over 18,000 annual ED visits and over 2000 inpatient beds. The ED provides care for patients triaged as level 1 or 2 based on the Emergency Severity Index (ESI) criteria [17]. Patients with ESI triage level 3 to 5 are taken care of in a separate urgency unit next to the ED, which is covered by general practitioners and internal medicine physicians. In our hospital, some patients with advanced illnesses are referred to the hospital’s palliative care clinic at the primary physician’s discretion on an outpatient basis. The clinic is operated by the palliative care team, a multidisciplinary team consisting of family physicians, nurses, and other healthcare professionals specializing in palliative care, as well as anesthesiologists specializing in pain management. They not only offer outpatient services at the palliative clinic but also operate a 4-bed palliative inpatient ward and provide consultant services for inpatients of other wards as well as ED patients during office hours on weekdays.

### Participants

Palliative patients over 18 years of age who visited the ED during the study period were prospectively enrolled. We defined palliative patients as those with at least one of the following conditions; 1) cancer of incurable stages or cancer with untreatable status, 2) organ failures affecting daily living, such as end-stage heart disease [18], decompensated liver cirrhosis [19], end-stage renal disease with no plans of renal replacement therapy, and end-stage lung disease (i.e., severe chronic obstructive pulmonary disease, severe pulmonary hypertension, pulmonary fibrosis) [20], and 3) neurodegenerative disorders, such as severe dementia, advanced-stage Parkinson’s disease, and previous stroke with totally dependent status. We also required that the primary treating physician of each included participant agreed with the palliative treatment decision.

### Ethics, study process, and data collection

The study protocol was ethically approved by the Siriraj Institutional Review Board (certificate no. 091/2019). All participants or their next of kin provided written informed consent prior to the study inclusion.

When eligible patients visited the ED, the ED attending physicians, who provided initial management to the patients, notified the study investigators of potential recruitment. The study investigators would wait until eligible patients were stabilized before initiating the enrollment and study process. After eligibility was confirmed and written consent obtained, a trained study investigator verbally interviewed each participant if their consciousness allowed and if they were not in any significant distress due to their conditions. Otherwise, the primary caregiver of the participant was the only interviewee. Data collected by means of an interview included patients’ and caregivers’ characteristics, while details of clinical manifestations, management, and outcomes were collected by using medical record review. The palliative performance score (PPS) was evaluated by interviewing the caregivers and having them assess the patients’ status prior to developing the symptoms that led them to the index ED presentation [21]. The main outcomes of the study were mortality in the ED and mortality at hospital discharge.

The study is reported according to the Strengthening the Reporting of Observational Studies in Epidemiology (STROBE) guideline [22].

### Statistical analysis

Descriptive statistics were employed to describe the patients’ and caregivers’ characteristics. Categorical data are reported as frequency and percentage. Continuous variables are reported as mean and standard deviation (SD) or median and interquartile range, as appropriate. Between-group comparisons were performed using the Chi-squared or Fisher’s Exact test for categorical data and an independent t-test or the Mann Whitney U test for continuous data. Characteristics were compared between patients discharged dead and alive and between those with and without cancer.

Multivariable logistic regression analyses were employed to assess independent factors associated with mortality in the ED and at hospital discharge. Factors included in the models were potential predictors of the outcomes based on literature review, scientific rationale, and univariable logistic regression analyses. They were age, sex, known case of cancer, documented advance care planning, previous palliative clinic consultation within 24 h before arrival, having been prescribed morphine within the index ED visit, palliative care team consultation in the index ED visit, and a PPS > 30 [21]. Independent factors associated with the outcome were chosen by using backward stepwise regression method. Results are reported as adjusted odds ratio (aOR) and its 95% confidence interval (CI). There were no missing outcomes. Although some variables in the models had missing data, the rate of missingness was negligible (< 5%); thus, they were judged to be missing completely at random, and no imputation techniques were attempted.

For all the analyses, a ***p*-**value of < 0.05 was considered statistically significant. All analyses were performed using SPSS version 18.0 (Chicago, IL., USA).

## Results

During the study period, 184 palliative patients visited the ED, 2 (1.1%) had died shortly after ED arrival and thus could not be recruited. Therefore, 182 patients were included in the study. The characteristics of the patients and their caregivers are presented in Tables 1 and 2, respectively. The patients’ mean age (± SD) was 73 ± 15 years, and 61.5% of them were female. Of all the patients, 98 (53.8%) had incurable cancer. Among cancer patients, 82.7% had metastatic cancer. On the other hand, neurodegenerative diseases were the most common cause of palliation in non-cancer patients (59%), followed by heart failure (8.2%), kidney failure (7.7%), and liver failure (4.9%). Most patients (74.1%) were brought to the ED based on the decision made by their relatives or nursing home staffs. Approximately 40% of all patients used emergency medical service as their mode of transportation to the hospital. Most patients visited the ED on weekdays (74.2%), and 52.2% came to the ED in the morning shift. Only 20.3% of included patients had previously visited the palliative clinic of our center.

**Table 1:** Patients’ characteristics

Characteristics	*N* = 182
**Age** (years)	73 ± 15
**Female sex**	112 (61.5)
**Marital status**	
Single	17 (9.3)
Married	109 (59.9)
Widowed	56 (30.8)
**Residence type** (missing = 20)	
Rented residence	27 (14.8)
Owner of residence	117 (64.3)
Nursing home	18 (9.9)
**Palliative care consultation before arrival**	6 (3.3)
**Cancer**	98 (53.8)
Ongoing treatment	10 (5.5)
Relapsed	7 (3.8)
Metastasis	81 (44.5)
**Acknowledgement of own disease(s)**	142 (78)
**Palliative performance score**	30 (10, 50)
**Primary chief complaint**	
Dyspnea	79 (43.4)
Altered mental status	32 (17.6)
Fever	18 (9.9)
Gastrointestinal symptoms	10 (5.5)
Bleeding	5 (2.7)
Genitourinary symptoms	4 (2.2)
Pain	3 (1.6)
Others	31 (17)
**Reason(s) of ED visit**	
Cannot control symptoms	146 (80)
Need to work-up other causes	66 (36.3)
No medical equipment at home	33 (18.1)
Cannot be left to die at home	28 (15.4)
Suggested by medical personnel	17 (9.3)
**Previous advance care plan** (missing = 2)	
Yes	110 (60.4)
Yes, with living will	9 (4.9)
Yes, with documented medical record	33 (18.1)
No	70 (38.5)
**Preferred place of dead** (missing = 3)	
Home	29 (15.9)
Hospital	17 (9.3)
Nursing home	1 (0.5)
No plan	132 (72.5)
**ED Interventions**	
Antibiotics	152 (83.5)
Bedside procedure, i.e., debridement, paracentesis	12 (6.6)
Inotropic drugs	20 (11)
Morphine	63 (34.6)
**ED primary diagnosis**	
Respiratory tract infection	72 (39.6)
Urinary tract infection	26 (14.3)
Progression of cancer	21 (11.5)
End-stage renal disease	3 (1.6)
Cardiovascular problem	8 (4.4)
Gastrointestinal problem	20 (11.0)
Sepsis	24 (13.2)
Others	8 (4.4)
**ED disposition**	
Admit inpatient ward	75 (41.2)
Admit palliative ward	18 (9.9)
Death	42 (23.1)
Discharge alive	32 (17.6)
Refer	15 (8.2)
**ED length of stay** (days)	2 (1,4)

*Note*: Data are presented as mean ± SD, median (25^th^, 75.^th^ percentile), or frequency (percentage)

*Abbreviations*: *ED* Emergency Department

**Table 2:** Caregivers’ characteristics

Characteristics	*n* = 182
**Age** (years)	50 ± 13.2
**Relation to patient** (missing = 1)	
Child	113 (62.1)
Wife/husband	24 (13.2)
Siblings	17 (9.3)
Grandchild	16 (8.8)
Daughter-/son-in-law	7 (3.8)
Parents	4 (2.2)
**Education** (missing = 2)	
Below primary school	15 (8.2)
Primary school	16 (8.8)
Secondary school	55 (30.2)
Bachelor’s degree	67 (36.8)
Master’s degree	25 (13.7)
Ph.D	2 (1.1)
**Occupation** (missing = 6)	
General employee	33 (18.1)
Private officer	41 (22.5)
Business owner	20 (11.0)
Agricultural worker	2 (1.1)
Trader	18 (9.9)
Government officer	26 (14.3)
Others	36 (19.8)
**Current work status** (missing = 2)	
Actively working	121 (66.5)
Did not previously work	43 (23.6)
Had to quit their work for patient care	16 (8.8)
**Understanding about the palliative status of the patient** (missing = 1)	
Yes	145 (79.7)
No	24 (13.2)
Not sure	12 (6.6)
**Understanding about planned management should the patient’ s symptoms worsen** (missing = 1)	
Yes	148 (81.3)
No	20 (11.0)
Not sure	13 (7.1)
**Preferred place of death for patient** (missing = 2)	
Home	42 (23.1)
Nursing home	4 (2.2)
Hospital	66 (36.3)
No plan	68 (37.4)

*Note*: Data are presented as mean ± SD or frequency (percentage)

The two most common chief complaints were dyspnea (43.4%) and altered mental status (17.6%). Commonly reported reasons for visiting the ED were ‘cannot control symptoms’ (80%) and ‘need to work-up other causes’ (36.3%). The majority of the patients (60.4%) had some form of advance care plan; however, only 4.9% had a living will and 72.5% had no plan regarding their preferred place of death. Among those with such plan, home was the most preferable location (15.9%). Additional patients’ characteristics are presented in Supplementary Appendix Table 1.

As for the caregivers, the patients’ child was the primary caregiver in 113 patients (62.1%). Most caregivers (66.5%) still worked full-time, while 8.8% had to quit their job. Approximately 80% understood the palliative treatment plan and knew the planned management had the patients gotten worse. However, 62.1% still had concerns about taking care of the patients at home. Discordant to the patients’ will, the most preferred place of death for the caregivers was the hospital (36.3%).

After ED arrival, the palliative care team was consulted by the ED physicians in 17% of the cases. Primary ED diagnoses mainly were related to infection, with respiratory tract infection as the most prevalent diagnosis (39.6%). Therefore, antibiotics administration was the most common management at the ED (83.5%). Although approximately half of the patients were admitted, only 9.9% were admitted to the palliative ward.

Of all palliative patients visiting the ED, 42 (23.1%) died in the ED, and 89 (48.9%) could not survive to discharge. Table 3 demonstrates the characteristics of patients stratified by mortality status at hospital discharge. Cancer was slightly more prevalent in those who died than in those discharged alive (*p* = 0.071). Non-survivors also received more palliative care consultation and morphine (both *p* < 0.001). On the contrary, survivors to discharge had a higher PPS (*p* = 0.025), and more of them decided to visit the ED by themselves (*p* = 0.035). We also analyzed independent factors associated with mortality (Table 4). Having received morphine and a PPS > 30 were significantly associated with mortality in the ED (aOR 4.05; 95%CI 1.81–9.03 and 0.27; 95%CI 0.1–0.7, respectively). While factors independently associated with in-hospital death were morphine, PPS > 30, cancer, and ED palliative consultation (aOR 4.92; 95%CI 2.24–10.81, 0.31; 95%CI 0.13–0.71, 2.62; 95%CI 1.07–6.41, and 3.88; 95%CI 1.23–12.22, respectively).

**Table 3:** Characteristics by mortality status at hospital discharge

Characteristics	Alive (*n* = 93)	Dead (*n* = 89)	*p*-value
**Age (years)**	73.7 ± 15.4	72.1 ± 15.3	.465
**Female sex**	57 (61.3)	55 (61.8)	.944
**Cancer**	44 (47.3)	54 (60.7)	.071
**Patients decided to visit the hospital themselves**	28 (30.1)	15 (16.9)	.035
**Patients acknowledged their diseases**	72 (77.4)	70 (78.7)	.841
**Patients’ preferred place of death**			.429
Home	16 (17.2)	13 (14.6)	
Nursing home	1 (1.1)	0 (0)	
Hospital	11 (11.8)	6 (6.7)	
**Caregivers’ plan on the patients’ place of death**			.411
Home	24 (25.8)	18 (20.2)	
Nursing home	2 (2.2)	2 (2.2)	
Hospital	28 (30.1)	38 (42.7)	
**Previous documented advance care plan**	68 (73.1)	72 (80.9)	.213
**Palliative consultation within 24 h before arrival**	2 (2.2)	4 (4.5)	.342
**Previous visit to the palliative clinic**	13 (14)	17 (19.1)	.352
**Palliative consultation in the ED**	6 (6.5)	25 (28.1)	< .001
**Morphine prescribed in the ED**	16 (17.2)	47 (52.8)	< .001
**ED length of stay < 2 days**	62 (66.7)	54 (60.7)	.401
**Palliative performance scale (PPS)**	42 ± 29	30 ± 22	.025*
**PPS > 30**	45 (48.4)	28 (31.5)	.020

*Note*: Data are presented as mean ± SD or frequency (percentage), *analyzed using the Mann–Whitney U test

*Abbreviations*: *ED* Emergency Department

**Table 4:** Factors independently associated with mortality from multivariable regression analyses

	**ED death (*n*** **= 41)**				**In-hospital death (*n* = 89)**			
	**Adjusted OR**	**95% CI**		***p*-value**	**Adjusted OR**	**95% CI**		***p*-value**
		**Lower**	**Upper**			**Lower**	**Upper**	
**Cancer**	1.37	0.54	3.49	0.512	2.62	1.07	6.41	0.035
**Morphine**	4.05	1.81	9.03	0.001	4.92	2.24	10.81	< 0.001
**PPS > 30**	0.27	0.1	0.7	0.007	0.31	0.13	0.71	0.006
**Palliative consult**	1.65	0.6	4.58	0.336	3.88	1.23	12.22	0.021

*Note*: Variables included in the model were age, sex, known case of cancer, documented advanced care planning, previous palliative clinic consultation within 24 h before arrival, having been prescribed morphine within the index ED visit, palliative care team consultation in the index ED visit, and a palliative performance score (PPS) > 30

*Abbreviations*: *ED* Emergency Department, *OR* odds ratio, *CI* confidence interval, *PPS* palliative performance score

When stratified the patients by the presence of cancer, we found that more cancer patients decided to visit the ED by themselves (*p* < 0.001) and more had previous palliative clinic visits (*p* < 0.001) than non-cancer patients (Table 5).

**Table 5:** Characteristics compared between cancer and non-cancer patients

Characteristics	Cancer (*n* = 98)	Non-caner (*n* = 84)	*p*-value
**Age** (years)	67.8 ± 14	78.9 ± 14.5	< 0.001
**Female sex**	55 (56.1)	57 (67.9)	0.13
**Decisions to visit the ED made by** (missing = 16)			< 0.001
Patients	34 (38.6)	9 (11.5)	
Relatives or nursing home staffs	54 (61.4)	69 (88.5)	
**Medical equipment at home**			
Nebulizer	6 (6.2)	16 (19)	0.01
Oxygen	22 (22.7)	32 (38.1)	0.02
Suction	1 (1)	21 (25)	< 0.001
**Previous palliative clinic visit**	29 (29.6)	1 (1.2)	< 0.001

*Note*: Data are presented as mean ± SD or frequency (percentage)

*Abbreviations*: *ED* Emergency Department

## Discussion

In Thailand, the palliative care system has not been well-established despite an increasing number of patients with incurable illnesses. This study describes the characteristics of palliative patients who visited the ED of the largest tertiary university hospital in Thailand. In the present study, approximately half of palliative patients did not have cancer but had other end-staged diseases, which was discordant with other studies in which most patients included were cancer patients [2, 4–7, 9–11]. Our finding indicates the need to expand the provision of palliative care and the field of palliative research to more extensive and diverse populations.

In many previous studies, dyspnea and pain were among the most common presenting symptoms that brought the patients to the ED [1, 2, 6, 23]. In this study, although we also found dyspnea as the most common chief complaint, pain was only reported in 1.6% of the patients. This discordance might have been because of effective home pain management provided by our healthcare providers and the palliative care team or due to the fact that our ED only provides care for very high-acuity patients triaged as level 1 or 2. While patients presenting with pain are usually triaged to level 3 to 5, and were thus sent to the urgency unit instead, thereby not being included in the present study. Moreover, we found that only a quarter of all patients decided to visit the ED by themselves, corresponding to a previous review stating that palliative patients, most of whom were cancer patients, often did not want to visit the ED [12]. However, in the present study, a significant number of non-cancer patients with neurodegenerative diseases and decreased cognitive ability were also enrolled, thus possibly underestimating the true prevalence of self-decision makers.

Also concordant with many previous studies, the main reason for ED visits in this study was that the patients could not control their symptoms [9, 10]. This could partly reflect the quality of our palliative system of care. In fact, palliative care recognition and utilization were immensely inadequate as only about 20% of the patients, most of whom were those with cancer, had previously visited our palliative clinic. Also, had there been a system of home consultation or home care services, ED visits due to this specific reason could have been reduced. Therefore, improving the quality of the palliative system and promoting its utilization, especially in non-cancer patients, are essential to enhance the well-being of end-of-life patients and reduce ED visits. Nonetheless, the rate of avoidable ED visits in this study was only 1.6% (data not shown), which was significantly lower than in other studies [4, 6, 7, 24], and the discharge rate was only 17.6%, demonstrating the very high-acuity and severity of the patients. Thus, it is questionable if improving the palliative care system will ever reduce ED visits to a large extent. Nevertheless, the lack of a standard system of palliative care might influence the quality of life of palliative patients in general, thus leading to, though unavoidable, unnecessary ED visits. Furthermore, a study by Verhoef MJ, et al. found that patients who had a proactive symptom management plan had lower in-hospital death (29.5%) than those without such plans [2]. Whereas in our study, only a handful of patients had those plans and even a smaller proportion truly understood them, corresponding to such a high rate of in-hospital mortality (48.9%).

The most common ED diagnosis was infection (67.1%), similar to a previous study [2] and concordant with the percentage of antibiotics administration. However, due to the lack of long-term care and hospices, the patients who required intravenous antibiotics had to be admitted. Nonetheless, the proportion of patients admitted to the hospital in this study was not different from other settings with long-term care and hospices implemented [5–7]. This paradoxical similarity was most likely because of the limited inpatient beds in our hospital, especially for palliative patients, as most of them had to stay in the ED. This crowding in the ED could also partly explain the high ED mortality rate in the present study [25].

Although approximately 60% of the patients had advance care plan, only 4.9% had a living will. This might have been because of Thai culture and tradition that limit the patients and families, who are the patients’ main caregivers, from discussing about advance care plan despite the patients having chronic diseases or cancer [26]. In fact, most of the main caregivers of the patients in the present study were their children, unlike a study by Lawson BJ, et al., from which the patients’ main caregivers were their spouses [13], who might have been more likely to discuss advanced care planning with the patients and have more influence on the family’s decision based on the Thai culture. Nonetheless, the families and physicians tended to choose the goals of treatment and advance care plan that they see fit and should meet the patients’ expectations [27]. However, our result showed that the patients preferred to die at home while their caregivers preferred otherwise. This finding was similar to a previous study in Thailand, which reported a diverse direction of perceptions between the patients and their caregivers [28]. Consequently, initiating palliative care as early as possible is fundamental to bring the patients and their caregivers together on the same page. The first step usually involves a discussion with all parties involved on the goals of care. Although initiating such an essential step in the ED has shown to provide benefits for the patients and lessen the burden of the health care system by reducing ED visits [1, 29], most emergency physicians were not comfortable doing so primarily because they did not have prior relationships with the patients and their families [2, 12]. Also, they did not consider themselves qualified in terms of knowledge and skills, and some also feared having conflicts with the patients’ families that may result in legal consequences [30]. Furthermore, the quality of this critical step performed in the ED may not be as desirable considering the time constraint in the ED setting.

We also found that having received morphine and a low PPS were associated with both ED and in-hospital death, concordant to a previous study [15]. However, cancer and palliative care team consultation in the ED were only independently associated with higher hospital mortality while failing to show significant associations with ED mortality. This discordance might have been because more patients with cancer were admitted, and very severe patients could have died in the ED before palliative consultation was initiated. In fact, the patients who had palliative care consultation could have been associated with higher in-hospital mortality because their symptoms were so severe, and they were judged to be approaching death, thus triggering the consultation for the purpose of both palliative ward admission and symptoms control. Regardless, these results emphasize the necessity of having a standardized and effective palliative care system aimed at both cancer and non-cancer patients. A specialized palliative care team and palliative care education for healthcare providers in the ED should also be encouraged.

## Limitations

Despite the study being one of the first among those conducted in suboptimal healthcare settings, the study had some limitations. First, it was a single-center study involving one tertiary university hospital, which may limit the study’s generalizability to other settings. The palliative care system in Thailand is different from other countries. With no standardized system, each region and hospital has their own distinct system, which may vary to a very large extent. Second, our ED only provides care for very high-acuity patients triaged as level 1 or 2, thereby underestimating the proportion of patients with unavoidable visits. Thirdly, we collected the data from the persons who came to the ED with the patients, some of whom might not have been the primary caregivers. Lastly, there were still some missing data from the questionnaire completed via in-person interview, which might have been caused by such a lengthy and detailed questionnaire. Further studies should thus emphasize on optimizing the format and length of the questionnaire and also the training of the interviewers.

## Conclusion

Approximately half of all palliative patients who visited the ED were non-cancer patients. The most common presenting symptom was shortness of breath, and the main reason for ED visits was an inability to control the symptoms. The patients and their caregivers still had limited and discordant understanding and perceptions. More importantly, the recognition and utilization of palliative care were largely inadequate, especially for patients without cancer. Therefore, an improvement and promotion in the palliative care system from the ED through home care as well as in-hospital services are mandatory to improve the quality of life of palliative patients.

## Supplementary Information

**Additional file 1:** **Table S1.** Additional patients’ characteristics.

## Data Availability

The dataset is not available but can be requested from the corresponding author.

## Abbreviations

ED: Emergency Department
ESI: Emergency Severity Index
PPS: Palliative performance score
SD: Standard deviation
OR: Odds ratio
CI: Confidence interval
