# Supplementary material for: Characteristics and factors associated with mortality in palliative patients visiting the Emergency Department of a large tertiary hospital in Thailand
Source: BMC Palliat Care. 2022 Jun 27;21:115. doi: 10.1186/s12904-022-01009-z (PMC9235171; doi:10.1186/s12904-022-01009-z)
Supplement: Supplementary file 1 — Additional file 1: Table S1. Additional patients’ characteristics. [file 12904_2022_1009_MOESM1_ESM.pdf]

Table S1. Additional patients' characteristics

Characteristics	N=182
**Religion**	
Buddhist	181 (99.5)
Christ	1 (0.5)
**Education** (missing =2)	
Below primary school	50 (27.5)
Primary school	59 (32.4)
Secondary school	39 (21.4)
Bachelor's degree	29 (15.9)
Master's degree	3 (1.6)
**Health coverage scheme** (missing = 1)	
Universal coverage	75 (41.4)
Social security scheme	4 (2.2)
State enterprise officer	93 (51.4)
Self-payment	9 (5)
**Number of family members at home**	4 (3,6)
**Household income** (Baht/month) (missing = 2)	
<9000 (275 USD)	13 (7.1)
9001-15000 (275-460 USD)	30 (16.5)
15001-30000 (460-920 USD)	49 (26.9)
30001-50000 (920-1530 USD)	39 (21.4)
50001-80000 (1530-2450 USD)	30 (16.5)
>80000 (2450 USD)	19 (10.4)
**Decision to visit the ED made by** (missing = 16)	
Patient	43 (25.9)
Relative or nursing home staff	123 (74.1)
**Mode of arrival**	
Own transport (i.e., own car, taxi)	106 (58.2)
Emergency medical service	76 (41.8)
**Previous palliative clinic visit**	37 (20.3)
**Frequency of palliative clinic visit**	
Every 1-3 months	30 (16.5)
Every 3-6 months	7 (3.8)
**Number of ED visits in the previous 6 months**	1 (0,3)
**Time of ED arrival**	
8:00-16:00	95 (52.2)
16:00-24:00	57 (31.3)
24:00-8:00	30 (16.5)
weekdays	135 (74.2)
**History of family death at home**	40 (22)
**Medical equipment at home**	
Nebulizer	22 (12.2)
Oxygen	54 (29.8)
Suction	22 (12.2)

Note: data are presented as median (25th, 75th percentile) or frequency (percentage)
Abbreviations: ED, Emergency Department
